# Health risk assessment of Patulin intake through apples and apple-based foods sold in Qatar

**DOI:** 10.1016/j.heliyon.2019.e02754

**Published:** 2019-11-21

**Authors:** Iman Saleh, Ipek Goktepe

**Affiliations:** Department of Biological and Environmental Sciences, College of Art and Science, Qatar University, P.O. Box 2713, Doha, Qatar

**Keywords:** Food science, Food microbiology, Hazard quotient, Daily intake, Health risk assessment, Patulin, Risk characterization

## Abstract

This is the first report on assessing the non-carcinogenic health risk associated with Patulin exposure in Qatar. The concentrations of Patulin, as determined in previous studies, in apples, apple juice, and apple-based baby foods sold in Qatar and nearby countries were used to conduct the health risk assessment (HRA). The risk related to Patulin intake by different age groups was calculated using the USEPA risk assessment models. The intake levels (ILs) of various age groups was compared with the international standards. The highest IL in Qatar was for babies between 5-12 months old through ingesting contaminated apple-based baby foods, yet those levels were below the tolerable daily intake of Patulin set by the EU at 0.4 μg/kg BW/d. The results showed that the intake of Patulin in Qatar is lower than that in Tunisia and Iran based on the HRA analysis. The risk caused by chronic exposure to Patulin through ingesting raw apples and apple juice separately was below “1,” indicating that the overall population is not likely to be at risk of Patulin exposure. However, various uncertainties should be considered when adopting these results, mainly the low number of samples and additive exposure to other mycotoxins from different sources.

## 1 Introduction

Patulin is a mycotoxin secreted by different fungal species, in particular, *Aspergillus, Byssochlamys* and *Penicillium*. It was first isolated from *Penicillium griseofulvum* in 1943 by Harold Raistrick (1943). When first discovered, Patulin was tested under the name of “tercinin” for its antimicrobial activity against gram positive and gram negative bacteria, however, it was not long until its toxicity was identified in 1944 (Chalmers and Clarke, 2004). *Penicillium expansum* is the main food contaminant known to produce Patulin. Patulin contamination occurs in a variety of food products, but apples are the most common fruits contaminated with this mycotoxin. Therefore, the concentration of Patulin in raw apples, as well as apple-based juices and food products is used as a quality indicator (Zhong et al., 2018). According to the World Health Organization (WHO), the maximum acceptable level of Patulin is set at 50 μg/L in apple juice, 50 μg/kg in solid apples, and 10 μg/L in kids and baby apple-based foods (EU, 2002; FDA, 2005; WHO, 2005). People get exposed to Patulin mainly through consuming infected food products. Fruit contamination might occur at different stages, including in field, during harvesting, at post-harvesting, during transportation, in stores, during display and throughout the processing stages if the produce is not sold raw (Tournas, 2005).

Mycotoxins related risks affect the entire population in geographic areas where food processing lack hygiene practices. High levels of Patulin affect all races, genders and age groups. Yet, some categories are more sensitive when it comes to exposure to toxicants. For instance, the level of Patulin in apple-based foods for kids has been set to be five times lower than the acceptable level for adults, which indicates that children below the age of 12 are a group of population at risk. Besides having higher exposure per kilogram body weight, children have different physiology that makes them more vulnerable (Raiola et al., 2015). Fetuses are even more sensitive population, as any exposure to toxins might affect their development; therefore, pregnant women and their fetuses are also at risk population (Chan-Hon-Tong et al., 2013). In addition, nursing infants are another at risk population, since breast milk might contain toxicants at levels higher than the tolerable daily intake (TDI) for this age group even if the mother is exposed to the adults TDI only (Degen et al., 2017).

Acute exposure to Patulin causes gastrointestinal symptoms including vomiting, nausea, ulcers, intestinal hemorrhages, and lesions in the duodenum (Kotsonis and Burdock, 2013). According to the International Agency for Research on Cancer (IARC), Patulin is a group 3 carcinogen, meaning that there is not enough animal research based studies or epidemiological data to support its carcinogenesis (IARC, 2018). Patulin's affinity to sulfhydryl groups has been widely described and its inhibitory effect towards many enzymes (ATPase, lysosomal enzymes, RNA polymerase etc.) has been explained (Puel et al., 2010). Nowadays, Patulin is known to be linked with neurological, gastrointestinal, and immunological adverse effects (Pal et al., 2017). In addition, the World Health Organization (WHO) considers Patulin as a possible genotoxic compound (WHO, 2005). Toxicity assessments of Patulin showed systematic adverse effects that damage vital organs including liver and kidney (Pal et al., 2017).

Health risk assessment analyses in the Middle East are rare, surveillance studies on Patulin levels are few, yet no exposure assessment evaluation was conducted based on the evaluated Patulin levels in this region. Therefore, this human health risk assessment study was carried out to determine the health risk associated with the ingestion of apple, apple juice, and apple-based-food products contaminated with Patulin for different age groups using the US Environmental Protection Agency (EPA) risk assessment models.

## 2 Methods

### 2.1 Data source and hazard identification

Data used in conducting HRA was extracted from existing literature as summarized in Table 1. The percentages of samples with contamination level above recommendations of WHO and EU were also calculated.

**Table 1:** The concentration of Patulin in various food products in Qatar, Turkey, Iran and Tunisia.

Sample type	Number of samples	Country	Contamination levels (μg/kg for solid and μg/L for liquid)	Average contamination level (+-SD)	Percentages of samples with contamination level above recommendations	reference
Apples	12	Qatar	ND-17.3	3.71 (0.6)	Adult level→ 0%	(Hammami et al., 2017)
					Children level → 16.6%	
Apple juice	20	Qatar	5.8–82.2	35.37 (1.66)	Adult level→ 25%	
Baby apple juice	6	Qatar	7.7–61.3	30.67 (6.7)	Children level→ 50%	
Baby apple compote	7	Qatar	1.02–24.57	10.92 (1.21)	Children level→ 42.8%	
Apple juice	36	Iran	0–190.7	52.8 (15.6)	Not calculated	(Rahimi and Rezapoor Jeiran, 2015)
Apple juice	30	Tunisia	0–167	80		(Zaied et al., 2013)
Mixed fruits juice	30	Tunisia	0–125	55	Adult level→ 18%	
Apple baby food	25	Tunisia	0–165	68	Children level→ 28%	
Apple juice	234	Turkey	5–376	63	Adult level→ 52%	(Gokmen and Acar, 2000)
Apple juice	119	Turkey	8–153	43	Adult level→ 34%	
Apple juice	67	Turkey	<5-103	19	Adult level→ 8%	
Apple juice	62	Turkey	<5-119	31	Adult level→ 8%	

### 2.2 Exposure assessment

Exposure assessment is the process of identifying the human population that could be at risk of contacting the contaminant of concern followed by estimation of the exposure level. As this assessment was intended to evaluate the human health risk associated with Patulin ingestion, chemical contamination levels and contaminated foods' consumption rates were first identified, then the daily intake of each of the assessed populations was calculated. Potential populations at risk included in this analysis were different age groups of children and adults. Pregnant women and nursing infants were not evaluated due to the lack of specific data on pregnant and nursing women’ apple and apple juice consumptions. The potential human exposure pathways were:

⁃ Ingestion of Patulin via raw apples or apple juice consumption.
⁃ Exposure to Patulin in apple-based baby foods.

#### 2.2.1 The consumption rates of fresh apples, apple juice, apple-based baby foods

According to the monitoring website Index Mundi the current Qatari population consumes around 20.000 metric ton of fresh apples per year (Index and Mundi, 2017).

All fresh apples sold in Qatar are imported from various countries, mainly from the Middle Eastern countries (Turkey Iran, Lebanon), USA, Oceana, and Europe (Family, 2015). Qatar exports fresh apples and produces its own apple juice and apple-based baby foods through two large factories located in the country (Kumar, 2018).

As the Qatari consumption data was not divided into different age groups based on each group's consumption rates, default consumption values established by the USEPA were used to calculate the Patulin daily intake values from ingesting apples and apple juice per μg/Kg BW/day.

Similarly, the USEPA default values were used to build the exposure assessment on Patulin intake of babies through the consumption of apple-based baby foods (EPA, 2011b). A conversion factor was used to convert the values from tablespoon to gram to calculate the Patulin daily intake, each tablespoon of baby food was considered to be equal to 15.94 g (Aqua-Calc, 2018).

The daily intake of Patulin through ingestion of fresh apples, apple juice, and/or apple-based baby foods (μg/kg/d) was calculated using the following formula (EPA, 1992):

$$\text{I}\left(\text{μg}/\text{kg}/\text{d}\right)=\frac{\text{C}\left(\text{μg}/\text{kg}\right)\times \text{AoF}\times \text{IGR}\left(\text{g}/\text{d}\right)\times \text{EF}\left(\text{d}/\text{yr}\right)\times \text{ED}\left(\text{yr}\right)\times \text{CF}\left(0.001\right)}{365\left(\text{days ​in ​a ​year}\right)\times \text{AT ​}\left(\text{yr}\right)\times \text{BW}\left(\text{kg}\right)}$$

I = Intake of Patulin (μg/kg bw/day)
C= Patulin contamination level in food (μg/Kg)
AoF = Oral absorption factor or bioavailability estimate (unitless)
IGR = Ingestion rate of food (g/day or L/day)
EF = Exposure frequency (days/year)
ED = Exposure duration (years)
CF = Conversion factor (used if the units in the formula do not match)
AT = Average time period of exposure (e.g. hours, days, months, years)
BW = Body weight of the assessed age group (kg)

According to the literature, various analyses showed different Patulin bioavailability values, most of the conducted studies are based on an artificial digestive system model known as *in-vitro* digestion (IVD). Assunçao et al. showed in 2016 that Patulin bioavailability ranged between 30 and 77% in artificially contaminated processed cereal samples (Assuncao et al., 2016). However, Raiola et al. have got values between 55 and 100% in apple sauce and baby fruits samples and have used 100% as Patulin bioavailability values in their health risk assessment (Raiola et al., 2012). Patulin bioavailability factor (AoF) was used in this study as “1” (T3DB, 2018). The Patulin daily intake was calculated in μg/Kg BW/day because the Patulin concentration in the Qatari samples was calculated in μg/Kg. The conversion factor (CF) used in this study was 10^−3^ to unify the unit between C and IGR. Exposure to many fruits and vegetables is considered as a daily based exposure and for those, the exposure factor ((EF x ED)/(365 x AT)) is equal to 1.

The final exposure model used to calculate the Patulin daily intake was:

$$\text{I}\left(\text{μg}/\text{kg}/\text{d}\right)=\frac{\text{C}\left(\text{μg}/\text{kg}\right)\times \text{AoF}\times \text{IGR}\left(\text{g}/\text{d}\right)\times \text{CF}\left(0.001\right)}{\text{BW}\left(\text{kg}\right)}$$

#### 2.2.2 Human body weight

According to Mundi index, children make 1/8^th^ of the Qatari population. The Qatari population age structure can be described as:

⁃ **0–14 years:** 12.63%
⁃ **15–24 years:** 12.35%
⁃ **25–54 years:** 70.59%
⁃ **55–64 years:** 3.42%
⁃ **65 years and over:** 1% (Index and Mundi, 2017).

According to Qatar Biobank report 2016/2017, the average weight of an adult male is 85.9 kg and the average weight of an adult female is 74 kg (Biobank, 2017). Since details of body weights of various age groups is not established yet, the default USEPA values were used in the exposure assessment model (EPA, 2011a).

### 2.3 Risk characterization

The chronic toxic risk caused by Patulin ingestion via apples, apple juice, and apple-based baby food was estimated using the formula described by (Torovic et al., 2018).

$$\text{HQ}=\frac{\text{I}\left(\text{μg}/\text{kg}/\text{d}\right)}{\text{PMTDI ​}\left(\text{μg}/\text{kg}/\text{d}\right)}$$

HQ = Hazard quotient
I = intake or potential exposure (e.g. μg/Kg BW/d)
PMTDI = Provisional Maximum Tolerable Daily Intake of Patulin set to 0.4 μg/kg BW/d (EC, 2006)

**Fig. 1:**
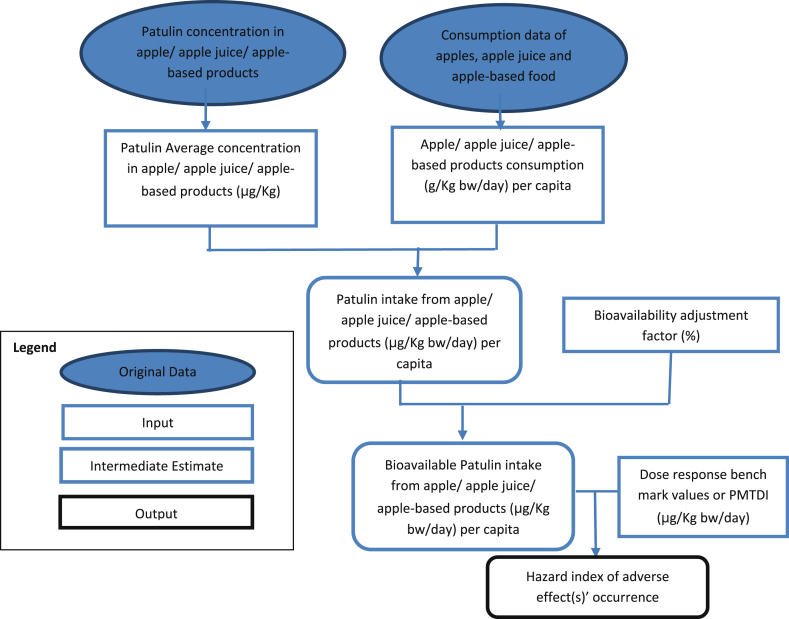
A model for risk characterization of Patulin exposure from apple, apple juice and apple-based foods.

A hazard quotient below “1” indicates a tolerable exposure level, while values higher than “1” indicates that adverse effect could occur and the exposure to the chemical requires further attention (Torovic et al., 2018). To estimate the hazard index of Patulin exposure from Qatari samples, the interrelationship of the risk assessment components was described in Fig. 1.

## 3 Results and discussion

### 3.1 Exposure assessment of Patulin (Qatar and nearby countries*)*

The Patulin daily intakes of various age groups through ingesting Qatari apple juice samples were summarized in Table 2. None of the age groups had a daily intake above the maximum tolerable daily intake of Patulin (PMTDI) set by the EU (0.4 μg/kg BW/day). Children below 6 years old were exposed to higher levels of Patulin compared to older age groups, making them the population at risk group.

**Table 2:** The Patulin daily intake values∗ for ingesting Qatari apple juice samples.

Age Group (years)	C (μg/Kg)	IGR ingestion rate (g/d)	BW (Kg)	I (μg/Kg/d)
0.5–1	30.67	14	9.2	**0.0467**
1–2	35.37	24	11.4	**0.0745**
2–3	35.37	27	13.8	**0.0692**
3–6	35.37	30	18.6	**0.0570**
6–11	35.37	24	31.8	**0.0267**
11–19	35.37	13	64	**0.0072**
>19	35.37	21	80	**0.0093**
References	(Hammami et al., 2017)	(EPA, 2011b)	(EPA, 2011a)	

∗ Bioavailability (AoF) is equal to 1 and the conversion factor (CF) is 0.001 (T3DB, 2018).

**Fig. 2:**
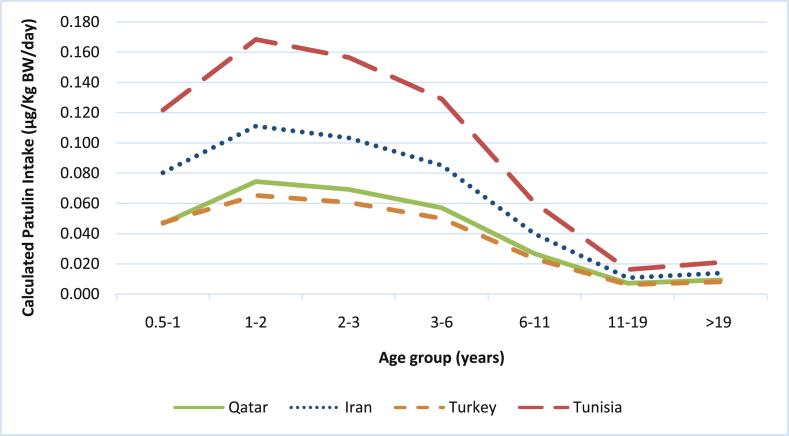
Mean per capita daily Patulin intake through apple juice consumption by different age group in Qatar, Iran, Turkey and Tunisia.

Patulin daily intakes of the same age groups were also calculated using data from nearby countries following the same calculation method. The countries assessed were Iran, Tunisia and Turkey. Although the Patulin daily intakes in Tunisia and Iran were higher than those in Qatar and Turkey, yet in all countries children below 6 years old were the most exposed. In addition, none of the age groups in the these three countries was exposed to a level above the PMTDI (Fig. 2).

For the calculation of Patulin daily intake through ingestion of Qatari apple samples, the ingestion rates of apples were used as per the 2003 EPA report (g/Kg BW/day). Table 3 summarizes the calculated intake values. The Patulin daily intake by consuming raw apple among different Qatari age groups is also lower than the recommended level.

**Table 3:** The Patulin daily intake∗ through consumption of raw apples sold in Qatar.

Age Group	C (μg/Kg)	IGR ingestion rate per BW (g/Kg-d)	I (μg/Kg/d)
6–12 months	3.71	9.7	**0.0360**
1–2 years	3.71	8.02	**0.0298**
3–5 years	3.71	4.103	**0.0152**
6–11 years	3.71	1.437	**0.0053**
12–19 years	3.71	0.582	**0.0022**
20–39 years	3.71	0.342	**0.0013**
40–69 years	3.71	0.357	**0.0013**
>70 years	3.71	0.434	**0.0016**
References	(Hammami et al., 2017)	(EPA, 2003)	

∗ Bioavailability (AoF) is equal to 1 (T3DB, 2018).

The Patulin daily intake of infants in Qatar via consumption of apple-based baby foods was calculated and summarized in Table 4. The Patulin intake level from baby food was within the acceptable limit, but it was the highest among the three sample types evaluated in Qatar.

**Table 4:** The Patulin daily intake through ingestion of baby apple compote samples sold in Qatar.

Age Group (months)	C (μg/Kg)	IGR ingestion rate (g/d)	BW (Kg)	I (μg/Kg/d)
4–5	10.90	58.978	6.6	**0.0974**
5–6	10.90	73.324	7.4	**0.1080**
6–12	10.90	89.264	9.2	**0.1058**
References	(Hammami et al., 2017)	(EPA, 2011b)	(EPA, 2011a)	

Apple-based baby food samples from Tunisia were analysed for their Patulin levels, the daily intake values were determined to be comparable to the levels of samples sold in Qatar. The calculated mean per capita daily Patulin intakes in Tunisia for the age groups 4–5 months, 5–6 months and 6–12 months are 0.608, 0.674, and 0.66 μg/kg BW/day respectively. The daily intake of Patulin through ingestion of apple-based baby foods sold in Tunisia was above the acceptable limit for the three at risk age groups and it is around six folds higher than the levels calculated for the same type of samples sold in Qatar. Many factors might play a role in this significant difference, such as the weather conditions, the countries of origin of apples, note that Tunisia grows some of its apples. It is worth noting that 25 samples were tested in Tunisia, while only seven samples were tested in Qatar. Therefore, more samples should be evaluated for an accurate assessment of Patulin contamination levels in apple-based baby food sold in Qatar.

### 3.2 Patulin daily intake in Qatari samples compared to worldwide data

The Patulin daily intake from Qatari samples ranged between 7.2 ng/kg bw/day and 74.5 ng/kg bw/day for apple juice samples, 1.3 ng/kg bw/day and 36 ng/kg bw/day for apple samples, and 97.4 ng/kg bw/day and 108 ng/kg bw/day for apple-based baby food samples. A review on the estimates of Patulin exposure for different age groups around the world was published by Marin et al. (2013). Foods containing apples were chosen to assess children and infant exposure to Patulin in nine different studies (Table 5). In all cases, the daily intakes calculated were below the Tolerable Daily Intake (TDI) values set at 400 ng/kg bw/day (Marin et al., 2013).

**Table 5:** Patulin exposure estimates of different age groups at various countries (Marin et al., 2013).

Country	Sample type	Number of samples tested	Population group	Exposure estimates (ng/Kg bw/day)	References
Belgium	Apple juice	177	Children	9–72	(Baert et al., 2007)
Italy	Apple-based product	169	Adolescents	1–14	(Piemontese et al., 2005)
France	Composites	20	Children	29–106	(Leblanc et al., 2005)
France	Composites	83	Children	1–97	(Sirot et al., 2013)
Netherlands	Apple-based product	-	Infants	17–307	(Brandon et al., 2012)
Catalonia (Spain)	Apple-based product	384	Infants	8–22	(Cano-Sancho et al., 2009)
Spain	Apple juice	100	Children	155	(Murillo-Arbizu et al., 2009)
South Africa	Apple juice	30	Children	1–37	(Shephard et al., 2010)
Sweden	Apple-based product	100	Children	5–24	(Thuvander et al., 2001)

The results obtained in this study for Qatari apple juice samples are very close to those calculated in Belgium and France (Table 5). However, the results of studies from South Africa had lower values, whereas the samples evaluated in Spain showed higher intake values. It is worth noting that Qatar imports all of its apples from mainly the USA and France; therefore, it is normal to have Patulin intake levels close to the ones obtained in France and other European countries.

A study conducted in Serbia between 2013 and 2015 on Patulin occurrence and risk assessment related to Patulin consumption by children, adolescence and adults via injestion of juice. The study showed a daily intake between 25.9 and 50.6 ng/kg bw/day for children and 2.8 and 5.5 ng/kg bw/day for adults. Serbian numbers are also below the TDI with the highest exposure for children (Torovic et al., 2018). In Romania, a study conducted on 50 samples of apple juice showed a daily intake of 53.2 ng/kg bw/day, which is in accordance with the daily intake obtained in our study (Oroian et al., 2014). An analysis conducted in China between 2006 and 2010 on 1987 apple juice samples showed that Patulin intakes for apple juice consumers among adults, children, and babies were estimated to be 28.1, 67.5, and 110 ng/kg bw/day, respectively (Guo et al., 2013). These results are similar to the results of Oroian et al. (2014) the highest exposure level was determined for babies (Guo et al., 2013). Patulin exposure was also assessed in Portuguese in 2016 among children consuming contaminated processed cereal-based foods. The daily intake values for Patulin among children below three years old were between 3.59 and 22.93 ng/kg bw/day, note that the intake values are lower than those encountered via apple-based food consumption (Assuncao et al., 2016).

### 3.3 Health risk assessment

**Fig. 3:**
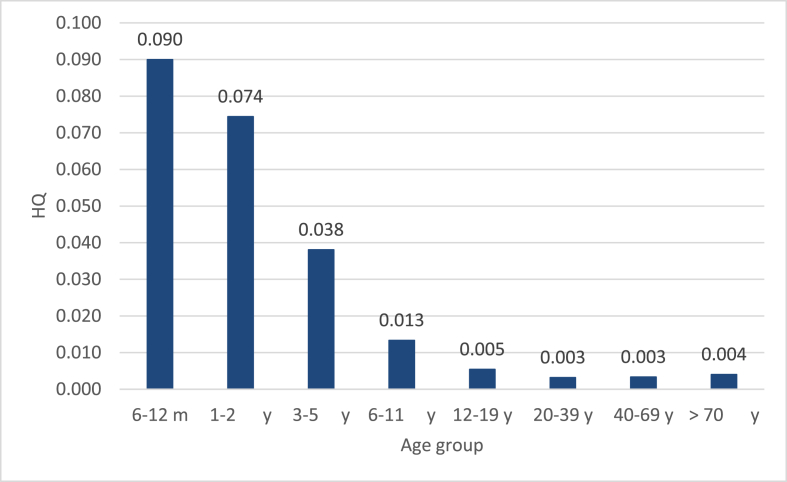
HQ of Patulin exposure via consumption of apples sold in Qatar.

**Fig. 4:**
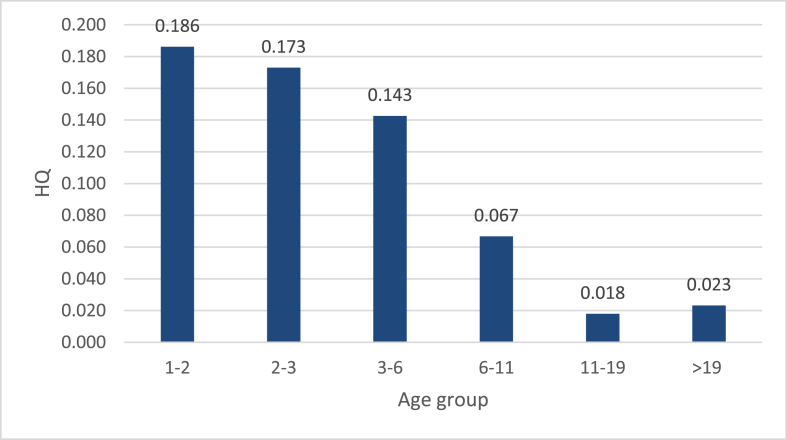
HQ of Patulin exposure via consumption of apple juice samples sold in Qatar.

The Patulin intake through consumption of both apples and apple juice was low for all age groups, which resulted in HQ ranging from 0.003 and 0.09 (Figs. 3 and 4). All HQ values were lower than “1,” indicating the unlikelihood of adverse effect occurrence via exposure to Patulin through apple-based samples' consumption in Qatar. The total hazard (HI) for consuming apples and apple juices sold in Qatar can be calculated by summing the HQ from the different sources of Patulin, HI represents the entire Qatari population's possible Patulin exposure risk. Although the HQ of each age group did show that the population is at risk, yet the sums, once different possible sources are investigated might exceed 1. It is important to emphasize that the consumption of apples, apple juice and other fruits and their juices takes place in parallel, meaning that an individual can get exposed to Patulin from different sources during a lifetime. Hence, the hazard indices (HI) from various sources could be added to provide the total hazard. This suggests that adverse effects linked to Patulin exposure for the overall Qatari population might be of concern, yet those estimates should be taken with cautions, as more data must be collected from the state of Qatar to come up with more precise estimates.

For Patulin exposure via apple-based baby foods available in Qatar, the HQ values range from 0.244 to 0.27. The HQ values for all age groups are below “1,” suggesting that the Patulin levels determined in apple-based baby food sold in Qatar is not likely to cause negative health effects among the Qatari population. However, it is important to note that the maximum tolerable level of Patulin used in the calculation was the same for adults and children. Nevertheless, the maximum allowed level of Patulin in apple juice for kids is 5 times lower than that of adults. If the same conversion factor would be used for the bench mark level used in the risk characterization calculations (PMTDI), the HQ values for most age groups would become higher than “1.” This implies that the younger age group requires further attention and a higher number of samples should be analyzed to determine children’ exposure Patulin via apple-based food consumption.

Compared to other risk characterization analysis around the world, a study conducted in Serbia showed also very low HQ related to consumption of Patulin via ingestion of contaminated apple juice, HQ of various age groups ranged from 0.007 to 0.127 using different consumption scenarios (Torovic et al., 2018). According to a study on Apple juice consumption in Romania, the HQ value for babies is 0.3 while HQ for older children and adults was 0.1, those results are different from ours where the highest HQ values were calculated for children between 1 and 6 years old (Oroian et al., 2014). In poorer communities, fruits are more prone to fungal infections, which is likely to increase mycotoxins occurrence. In Pakistan, a study conducted on 237 samples showed HQ value related to fruits’ consumption by the entire population of 1.22, which means that people in Pakistan are at risk of Patulin adverse health effects via fruits ingestion. As for the HQ related to the consumption of juices and smoothies, values were calculated at 0.4 and 0.35, respectively. The findings of the Pakistani study are relatively different from the results obtained in this study, as Qatari raw fruits showed a lower Patulin exposure risk than those of juice samples, yet it is worth noting that this study concentrated on apples only, while the Pakistani study included various pome fruits (Iqbal et al., 2018).

### 3.4 Uncertainty assessment

In the risk assessment studies, uncertainty assessment should be conducted by the end to evaluate the main assumptions of the study and to draw meaningful conclusions (Yost et al., 2017). The uncertainties considered in this study include:

• The low number of samples surveyed to determine Patulin concentration in apples, apple juice, and apple-based baby food samples sold in Qatar was the major issue.
• County of origin of the samples sold in Qatar was not indicated, making the comparison difficult.
• The default values used in the exposure assessment were based on the EPA surveys. The food intake might differ among countries; therefore, an actual survey should be conducted in the state of Qatar.
• The bioavailability assumption made in this study could be an over-estimation as it is unlikely to have all the Patulin ingested are completely metabolized. Numbers in the literature varies yet many risk assessment reports have assumed that bioavailability in the digestive system is equal to 100%
• The lack of data for masked Patulin levels and its fate in the human body are other uncertainties. Patulin might bind to the solid parts of the apple in the juice, forming “masked Patulin” which cannot be experimentally detected. Although “masked Patulin” is not toxic on its own, it might be hydrolysed in the digestive system of the mammals back to its toxic form (Berthiller et al., 2013). Therefore, the detected levels of Patulin in food samples might be an underestimation of its actual concentration.

## 4 Conclusions

The evidence collected from literature review suggests that people around the world might be at risk of Patulin exposure. Although data on Patulin levels in Qatari samples is limited, it was determined that many of the apple-based samples tested exhibited Patulin concentrations higher than the acceptable limit, indicating that Qatar might be one of the geographic areas at risk. According to the risk characterization analysis conducted in this study, Patulin exposure is not likely to cause adverse health effects in the Qatari population. However, more samples should be evaluated in future studies to draw more meaningful conclusions. A special attention should also be given to children’ exposure as they are the most sensitive population at risk of Patulin exposure. The chemical analysis research should concentrate on extracting and determining the masked Patulin levels, and biological simulation models should focus on the fate of this assumed none-toxic form of Patulin in the human digestive system. Finally, carefully planned surveillance studies to determine Patulin exposure through consumption of other pome fruits and their products should be carried out.

## Declarations

### Author contribution statement

Iman Saleh: Performed the experiments; Analyzed and interpreted the data; Wrote the paper.

Ipek Goktepe: Conceived and designed the experiments; Analyzed and interpreted the data; Contributed reagents, materials, analysis tools or data.

### Funding statement

The publication of this article was funded by the Qatar National Library.

### Competing interest statement

The authors declare no conflict of interest.

### Additional information

No additional information is available for this paper.
